# Systolic Blood Pressure Variability When Transitioning From Intravenous to Enteral Antihypertensive Agents in Patients With Hemorrhagic Strokes

**DOI:** 10.3389/fneur.2022.866557

**Published:** 2022-07-01

**Authors:** Abdulrahman I. Alshaya, Meshari Alghamdi, Sumaya N. Almohareb, Omar A. Alshaya, Mohammed Aldhaeefi, Abdullah F. Alharthi, Sulaiman Almohaish

**Affiliations:** ^1^Department of Pharmacy Practice, College of Pharmacy, King Saud bin Abdulaziz University for Health Sciences, Riyadh, Saudi Arabia; ^2^King Abdulaziz Medical City, National Guard Health Affairs, Riyadh, Saudi Arabia; ^3^King Abdullah International Medical Research Center, Riyadh, Saudi Arabia; ^4^Department of Clinical and Administrative Pharmacy Sciences, College of Pharmacy, Howard University, Washington, DC, United States; ^5^Department of Pharmacotherapy and Outcomes Science, School of Pharmacy, Virginia Commonwealth University, Richmond, VA, United States; ^6^Pharmacy Practice Department, College of Clinical Pharmacy, King Faisal University, Hofuf, Saudi Arabia

**Keywords:** intracerebral hemorrhage, subarachnoid hemorrhage, antihypertensive, blood pressure, neurocritical care

## Abstract

**Background/Objective:**

Systolic blood pressure variability (SBPV) in patients with intracranial hemorrhage (ICH) and subarachnoid hemorrhage (SAH) is associated with an increased risk of acute kidney injury (AKI) and mortality. SBPV is a strong predictor of poor functional outcomes in patients with ICH. Intravenous (IV) antihypertensive agents are commonly used to achieve sustained target blood pressure goals; however, this is not a feasible long-term option. The transition from IV to enteral antihypertensives is not yet well established in patients with ICH and SAH. This study aimed to assess the effect of the number of antihypertensive agents and overlap time during the transition period from IV to enteral route on SBPV in patients with ICH and SAH.

**Methods:**

This retrospective single-center study was conducted at a tertiary teaching hospital in Riyadh, Saudi Arabia. Data were extracted from electronic medical records after obtaining Institutional Review Board approval. Patients were included if they were >18 years old, admitted with spontaneous ICH or SAH, and received continuous infusion antihypertensives prior to transitioning to the enteral route. The major outcome was the effect of the number of antihypertensive agents and overlap time on SBPV during the transition process. Minor outcomes included the effect of the number of antihypertensive agents and overlap time on heart rate variability and the incidence of AKI on day 7.

**Results:**

After the screening, we included 102 patients. Based on our regression model, the number of enteral antihypertensive agents upon transitioning from IV to enteral antihypertensive therapy had no effect on SBPV in the intensive care unit (ICU) among our patients (*p*-value = 0.274). However, the prolonged overlap was associated with reduced SBPV in the ICU (*p*-value = 0.012). No differences were observed between the groups in heart rate variation or AKI rate.

**Conclusions:**

In patients with ICH and SAH, prolonged overlap of enteral antihypertensive agents to overlap with intravenous antihypertensive therapy may result in lower SBPV. This finding needs to be confirmed on a larger scale with more robust study designs for patients with ICH and SAH.

## Introduction

Hemorrhagic stroke represents 10−20% of stroke cases annually (1). It is considered a neurological emergency that requires complete and comprehensive management as it carries a high risk of mortality and morbidity (2, 3). Acute hypertension is a common finding in patients with intracranial hemorrhage (ICH) or subarachnoid hemorrhage (SAH) (4). High blood pressure is an independent cause and risk factor for hemorrhagic stroke, and significant systolic blood pressure variability (SBPV) in these patients may lead to poor functional status and mortality (5–8). The exact mechanism and etiology of this variability are still not fully understood. However, it could be related to either an increase in the sympathetic system activity, reduced baroreceptor reflexes, increased arterial stiffness, humanteral, rheological, environmental, behavioral, and emotional factors, age, activity/sleep, and medication-related issues (9, 10). The optimal management of blood pressure (BP) during the acute stages of hemorrhagic stroke remains controversial. Available data from large randomized trials did not provide consistent recommendations regarding target BP and how to achieve it. The American Heart Association guidelines for ICH and SAH recommend targeting a systolic BP goal of <140–160 mmHg during the acute phase (11, 12).

The magnitude of safe BP reduction from baseline BP upon admission with ICH or SAH has not yet been established. BP fluctuation or variability in ICH or SAH has been discussed in multiple recent studies that examined SBPV during the first 24 h in patients with ICH or SAH (13, 14). They found an association between SBPV and unfavorable functional outcomes. The *post-hoc* analysis of the Intensive Blood Pressure Reduction in Acute Cerebral Hemorrhage Trial 2 (INTERACT2), Field Administration of Stroke Therapy-Magnesium (FAST-MAG), and Antihypertensive Treatment of Acute Cerebral Hemorrhage II (ATACH-2) trials found that SBPV is a strong predictor of poor outcomes in patients with ICH and increases mortality risk with worse neurological outcomes (13–15). Regarding patients with SAH, current evidence provides similar results regarding SBPV and poor outcomes, as in patients with ICH (8, 16).

Intravenous (IV) antihypertensive agents, such as labetalol and nicardipine IV continuous infusion, are commonly used to achieve sustained target BP goals in the acute phase of hemorrhagic stroke (17, 18). Nonetheless, this is not a feasible, long-term option. The transition process of antihypertensive agents from IV to an enteral route is not yet well established in patients with ICH or SAH. Thus, this study aimed to investigate the SBPV of antihypertensive agents during the transition period from IV to enteral route in patients with ICH and SAH.

## Methods

This retrospective cohort study was conducted at King Abdulaziz Medical City, National Guard Health Affairs (NGHA), which is a tertiary teaching hospital in Riyadh, Saudi Arabia. Data were obtained from electronic medical records (BESTCare) for all patients admitted with a neurocritical illness for at least 24 h from January 1, 2016 to December 1, 2020. Institutional Review Board approval for this study was granted by the King Abdullah International Medical Research Center (KAIMRC) with study number RC20/578/R. Patients were included if they had a documented diagnosis of ICH or aneurysmal SAH. Patients should have received continuous IV BP agents at any time during ICU admission and be aged >18 years prior to being included in the study. Patients were excluded if they had a traumatic ICH/SAH or if they had a documented shock state including septic shock, hemorrhagic shock or cardiogenic shock the time of BP agent administration. We exclude patients with shock states to minimize factors that could affect blood pressure variability.

Demographic data collected at baseline included age, sex, body mass index, serum creatinine (μmol/L) upon ICU admission, creatinine clearance based on the Cockcroft-Gault equation upon ICU admission, history of chronic hypertension and/or diabetes, ICH score upon admission, and Fischer score. Surgical history, including decompressive craniectomy/craniotomy, external ventricular drainage (EVD), and Jackson-Pratt drain placement, was also collected. We screened for patients who were prescribed IV BP agents, including labetalol or nicardipine, during their hospital stay. Additionally, all relevant co-administered agents that could affect BP were collected, including adjunctive IV or enteral antihypertensive agents, propofol, dexmedetomidine, and intravenous opioids. Information including IV BP agents, average dose over the last 24 h, time from admission to overalp to enteral BP agent(s), overlapping time with enteral agent(s), and rate of IV agent re-initiation. Systolic blood pressure (SBP) goals and agents used as needed for BP management were collected. Safety data included delta in SBP for the first 24 h after the overlap from IV BP agents to enteral BP agents, which was calculated by subtracting the minimum and maximum SBP readings (delta in SBP = minimum SBP–maximum SBP). The major outcomes included delta in SBP as an indicator for SBPV. Minor outcomes included the incidence of hypotension (SBP <90 mmHg), bradycardia (heart rate [HR] <50 beats per min), the incidence of acute kidney injury per Acute Kidney Injury Network criteria on day 7, and the number of IV BP agents used during the first 24 h after transition, and the need for enteral agent up-titration or addition of other BP-lowering agents. Moreover, ICU length of stay (LOS) and hospital LOS were also reported as minor outcomes. Systolic blood pressure titration at our institution usually starts when nicardipine is down trending below 5 mg/hr or labetolol below 4 mg/min, indicating a reasonable point to initiate the transition process. Our intensivists mostly prescribed a dihydropyridine calcium channel blocker (CCB), angiotensin-converting enzyme inhibitor (ACEI), or angiotensin-receptor blocker to overlap with IV antihypertensive therapy and safely transition patients with ICH and SAH to their ultimate enteral regimens. Sample size calculations were not performed prior to the study but rather we screened all ilegible patients to reduce potential selection bias. The baseline characteristics of the participants were presented as mean ± standard deviation or median (interquartile range [IQR]) for continuous variables and numbers (proportions) for categorical variables, as appropriate. Continuous data were analyzed using the Student's *t*-test or the Mann-Whitney U test. Chi-square and Fisher's exact tests were used to compare categorical data as appropriate, and statistical significance was defined as *p* < 0.05.

Descriptive statistics were used to summarize patient demographics and clinical outcomes, with data presented as medians [IQR1–IQR3] and frequencies (percentages), as appropriate. We used a multiple linear regression model to predict the SBPV in the ICU (major outcome) based on two independent variables: the number of enteral antihypertensive agents while transitioning from IV to enteral antihypertensive therapy and the overlapping time between IV and enteral antihypertensive agents. Subsequently, we used the same model to predict HR variability in the ICU as a minor outcome. Regarding other minor outcomes, the Mann-Whitney U test was used to compare continuous variables, and the Pearson chi-squared test was used to compare categorical variables. The unadjusted odds ratios (ORs) of minor outcomes and the associated 95% confidence intervals (CI) were calculated using the Baptista-Pike method. The Pearson correlation test was performed to determine the relationship between SBP and HR variabilities and the number of enteral agents used upon transitioning. Pearson correlation coefficients were interpreted as follows: >0.8, very strong; 0.6–0.8, strong; 0.4–0.6, moderate; 0.2–0.4, weak; and <0.2, negligible correlation. Statistical significance was defined as 2-sided *p*-values < 0.05. Statistical analyses and data graphing were performed using Prism 9 version 9.2.0.

## Results

Of the 125 patients screened, 102 were included in this study. The baseline demographics are shown in Table 1. Most of our patients (59.8%) had aneurysmal subarachnoid hemorrhage (aSAH) as their primary diagnosis. Other management strategies, such as decompressive craniectomy, EVD placement, and Jackson-Pratt drain, were used in only 10 (9.8%) patients. Only 19 (31.15%) and 14 (34.15%) patients with aSAH and ICH received enteral antihypertensive therapy before ICU admission, respectively.

**Table 1:** Baseline characteristics.

**Characteristic**	**aSAH patients**	**ICH patients**
	**(*n* = 61)**	**(*n* = 41)**
Age, years*	58 [45–68.5]	61 [46.5–74.5]
Gender, male^#^	47 (77.05)	37 (90.24)
Weight, kg*	80 [74–94.3]	75 [68.6–83]
SCr, mmol/L*	83 [66.5–111.5]	85 [68.25–116.75]
Comorbidities^#^		
HTN	47 (77.05)	33 (80.49)
DM type 2	34 (55.74)	22 (53.66)
SBP during the first 24 h, mmHg*	166 [151.5–182.5]	164 [149–180.25]
ICH score*	NA	1 [0–2]
Management strategies^#^		
Decompressive craniectomy	0 (0)	0 (0)
EVD placement	0 (0)	8 (19.51)
Jackson-Pratt drain	1 (2.44)	1 (2.44)
On antihypertensive medication before admission^#^	19 (31.15)	14 (34.15)
Oral antihypertensive medications used during transition^#^	137	86
Calcium channel blockers	48 (35)	36 (41.8)
Angiotensin converting enzyme inhibitors	31 (22.6)	20 (23)
Angiotensin II receptor blockers	9 (6.5)	5 (0.05)
Beta-blockers	24 (17.4)	11 (12.7)
Vasodilators	25 (18.1)	13 (15.1)
Diuretics	–	1 (0.01)

*Median [IQR1-IQR3]; ^#^n (%); aSAH, aneurysmal subarachnoid hemorrhage; DM, diabetes mellitus; ICH, intracerebral hemorrhage; HTN, hypertension; SBP, systolic blood pressure; SCr, serum creatinine; EVD, external ventricular drain; NA, not applicable.

### Major Outcome

**Figure 1:**
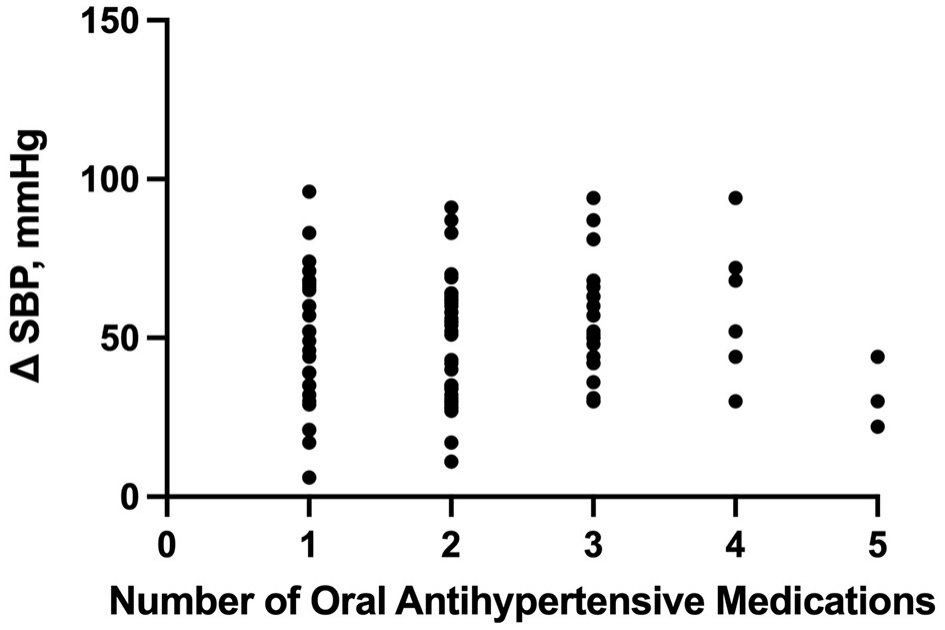
Correlation between the number of enteral (PO) agents and systolic blood pressure delta. PO, per os (enteral); SBP, systolic blood pressure.

**Figure 2:**
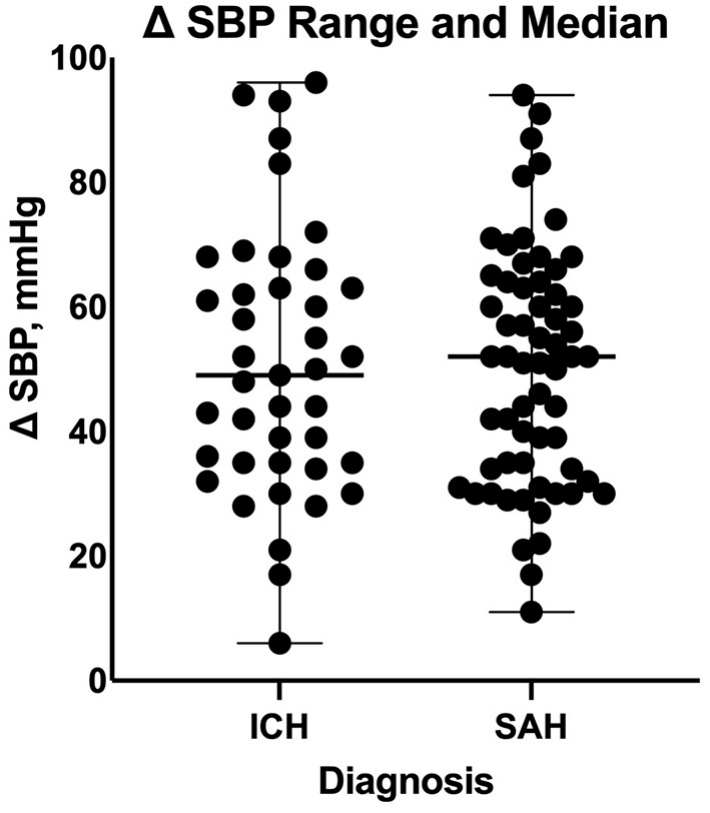
Comparison of systolic blood pressure delta between patients with intracranial hemorrhage and those with subarachnoid hemorrhage during the transition process. ICH, intracranial hemorrhage; SAH, subarachnoid hemorrhage; SBP, systolic blood pressure.

Based on our multiple linear regression model, the number of enteral antihypertensive agents upon transitioning from IV to enteral antihypertensive therapy had no effect on the SBPV in the ICU among our patients (*p*-value=0.274, 95% CI−1.751–6.095). The overlap time between IV and enteral antihypertensive therapy had a statistically significant effect on SBPV in the ICU (*p*-value=0.012, 95% CI−3.652-−0.448). Every 2.051 h of overlap between the IV and enteral antihypertensive therapy while transitioning resulted in reduced SBPV by 1 mmHg among our patients. The median time for overlap was 3 days [Q1–Q3: 2–5]. The SBPV data are shown in Figures 1, 2.

On a separate analysis for each disease cohort based on our multiple linear regression model, the overlap time between IV and enteral antihypertensive therapy had a statistically significant effect on SBPV among ICH patients only (*p*-value = 0.0054, 95% CI −8.199 −1.533). Every 4.866 h of overlap between the IV and enteral antihypertensive therapy of overlap resulted in reduced SBPV by 1 mmHg among our ICH patients. The overlap time and number of enteral antihypertensive agents had no effect among SAH patients.

### Minor Outcomes

**Figure 3:**
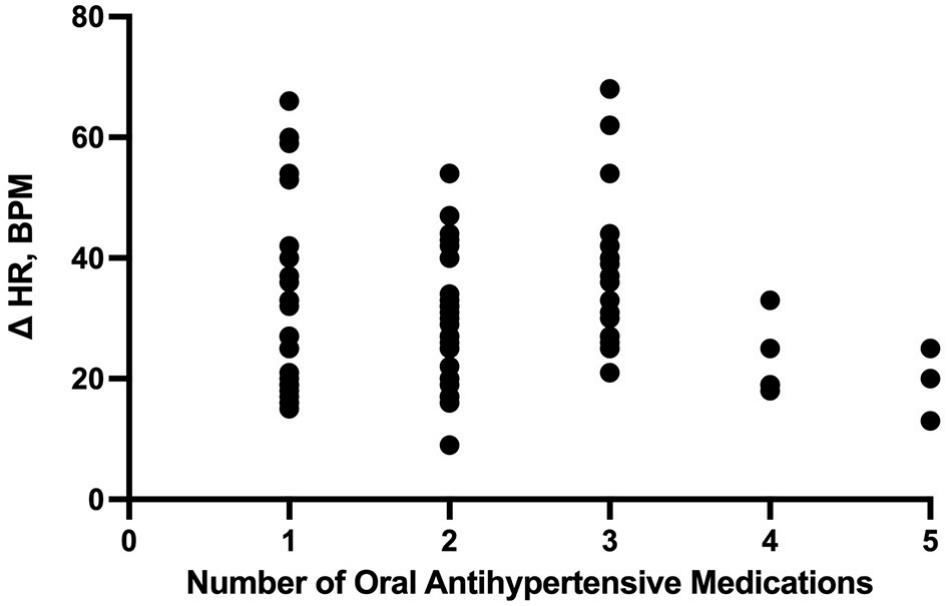
Correlation between the number of enteral (PO) agents and heart rate delta. HR, heart rate; PO, per os (enteral).

**Figure 4:**
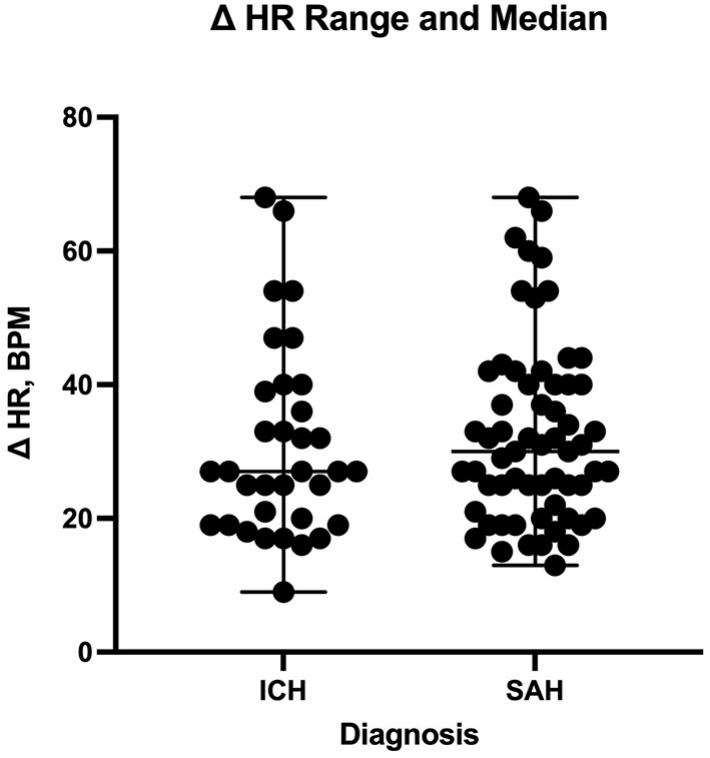
Comparison of heart rate delta between patients with intracranial hemorrhage and those with subarachnoid hemorrhage during the transition process. HR, heart rate; ICH, intracranial hemorrhage; SAH, subarachnoid hemorrhage.

The number of enteral antihypertensive agents upon transitioning from IV to enteral antihypertensive therapy (*p*-value = 0.675, 95% CI −3.386–2.204) and overlap time (*p*-value = 0.653, 95% CI −1.433–0.902) between IV and enteral antihypertensive therapy did not affect HR variability among our patients in the ICU. There was a positive negligible correlation between the number of enteral antihypertensive agents used and SBPV (r = 0.035, *p* = 0.728, 95% CI −0.161–0.228). Nonetheless, we found a negative correlation between the number of enteral antihypertensive agents used and HR variability which was not statistically significant (r = −0.059, *p* = 0.57, 95% CI −0.258–0.145). The data for HR variability are shown in Figures 3, 4.

Table 2 summarizes other secondary outcomes. Based on our definition of “major SBPV” in the ICU, 83 (81.37%) patients met our definition and had an SBPV of >30 mmHg. The other 19 (18.62%) patients had SBPV of ≤ 30 mmHg. Mortality rates were similar between the two groups (OR 0.603, 95% CI 0.05–3.585, *p*-value = 0.642). Similarly, patients had a similar rate of AKI during their ICU stay (OR, 0. 0.827, 95% CI 0.251–2.555, *p* = 0.749), regardless of SBPV. However, patients with SBPV >30 mmHg had a shorter ICU LOS with a median of 6 [2–14] days (*p* = 0.005, 95% CI 2–10), as compared to 14 [9.75–16] days for patients with SBPV ≤ 30 mmHg. There was no statistically significant difference between the two groups in the 7-day HR <50 bpm events (OR 0.631, 95% CI 0.18–2.233, *p*-value = 0.631). Conversely, patients with SBPV ≤ 30 mmHg had higher 7-day SBP <90 mmHg events with a rate of 42.1% vs. 13.25% for those with an SBPV of > 30 mmHg (OR 4.76, 95% CI 1.574–15.01, *p* = 0.003).

**Table 2:** Comparison of outcomes between patients with SBP variability of ≤ 30 mmHg vs. > 30 mmHg.

	**≤30 mmHg patients (*n* = 19)**	**> 30 mmHg patients (*n* = 83)**	**Odds ratio**	**p-value**	**95% confidence interval**
AKI^#^	4 (21)	15 (18.1)	0.827	0.749	0.251–2.555
ICU mortality^#^	1 (5.26)	7 (8.43)	0.603	0.642	0.05–3.585
ICU length of stay, days*	14 [9.75–16]	6 (2-14)	N.A	0.0049	2–10
HR <50, bpm^#^	3 (15.79)	19 (22.89)	0.631	0.757	0.18–2.233
SBP <90, mmHg^#^	8 (42.1)	11 (13.25)	4.76	0.003	1.574–15.01

*Median [IQR1-IQR3]; ^#^n (%); AKI, acute kidney injury; HR, heart rate; ICU, intensive care unit; SBP, systolic blood pressure.

## Discussion

Our study is the first to evaluate the effect of enteral antihypertensive overlap with IV therapy on SBPV in patients with ICH or SAH. The results from this study show that prolonged overlap antihypertensives overlapping with IV antihypertensives can significantly lower SBPV in the ICU. In addition, this study showed that every 2 h of overlap between IV and enteral antihypertensives can lower the SBPV by 1 mmHg, which indicates that prolonged overlap enteral antihypertensive initiation could result in lower SBPV. SBPV was associated with an increased risk of in-hospital mortality using five different indices, including SBP “range” or “delta,” which is the difference between the maximum and minimum SBP readings (> 50–60 mmHg) (19). In the subgroup analysis, an SBP delta <30 mmHg was associated with better outcomes. The ORs for poor outcomes were 5.2 and 9.0 for SBP delta of 29–44 and >44 mmHg, respectively, in patients with SAH (16).

In our study, HR variability was not affected by the overlap time or number of enteral antihypertensives used, which might be related to the class of antihypertensive agents used during this process. Previous studies have investigated SBPV using IV antihypertensive agents in patients with ICH and SAH (13–15, 20–22). In a study by Liu-DeRyke et al., 28 patients on labetalol were compared to 26 patients receiving nicardipine. They concluded that patients receiving nicardipine had lower SBPV than patients receiving labetalol to control their BP (17). In the contrary, it is unclear why patients who developed higher SBPV in our cohort had shortened ICU length of stay despite using similar utilization of IV antihypertensive agents. Nonetheless, Poyant et al. compared nicardipine ± labetalol and/or hydralazine therapy with labetalol and/or hydralazine. They found that patients who received a nicardipine-containing regimen had lower SBPV than those who received labetalol and/or hydralazine-containing regimens (18). This might be because nicardipine has a quick on/off onset of action, which makes its titration quicker than labetalol. A study by Zhu et al. found that early initiation of oral antihypertensive therapy in patients with ICH may reduce nicardipine use and ICU LOS. Their outcome may potentially reflect a potential role for early introduction of oral antihypertensive agents to offer cost saving opportunities, with a median number of three antihypertensive agents at discharge (23). In our cohort, the number of administered antihypertensive agents did not result in less SBPV and did not affect the ICU LOS. The reported AKI rate in this study was 21% in the included cohort, which is similar to our cohort (23). It is essential to interpret this rate carefully as many variables may lead to this outcome, such as baseline hypertension, significant hypotension, the use of nephrotoxic agents, or the use of certain antihypertensive agents, such as ACEIs compared to CCBs (24). Additionally, it may be essential to consider the pharmacokinetic aspects of the selected antihypertensive agent in this population in terms of the onset of action during the overlap process with IV antihypertensive agents. Oral agents including ACEIs such as lisinopril may have a quicker onset, approximately 6 h, compared to dihydropyridine CBBs, such as amlodipine (approximately 24–49 h) (25). Future prospective studies may be needed to compare several enteral antihypertensive regimens, such as ACEIs compared to CCBs, during the transition period in patients with ICH and SAH.

There were a few limitations associated with our study, including the relatively small sample size. However, our study is the first to evaluate the effect of enteral and IV antihypertensive overlap on SBPV. Moreover, the study design as a single-center retrospective study may limit the generalizability of our findings. The insufficient documentation of JP drain and EVD was a challenge to capture the accurate number of its utilization amongst our patients. Documentation of Hunt and Hess and Fischer scoring tools was lacking, in addition to the inconsistent documentation of Glasco Coma Scale which could have been added value to estimate clinical outcomes of the included SAH patients. Our median ICH score of 1 may limit the generalizability of our outcomes to more severe ICH cases. Despite these limitations, which were mainly driven by our study's exploratory nature, our findings may open the path for future large prospective trials to optimize the transition process in patients with ICH and SAH.

## Conclusions

In patients with ICH and SAH, the prolonged overlap of enteral antihypertensive agents may result in lower SBPV. The number of enteral antihypertensive agents used during the transition process did not correlate with lower SBPV. These findings need to be confirmed on a larger scale with more robust study designs for patients with ICH and SAH.

## Data Availability Statement

The raw data supporting the conclusions of this article will be made available by the authors, without undue reservation.

## Ethics Statement

This study was approved by the Institutional Review Board of the King Abdullah International Medical Research Center (KAIMRC) with study number RC20/578/R. All procedures followed have been performed in accordance with the ethical standards laid down in the Declaration of Helsinki.

## Author Contributions

All authors listed have made a substantial, direct, and intellectual contribution to the work and approved it for publication.

## Conflict of Interest

The authors declare that the research was conducted in the absence of any commercial or financial relationships that could be construed as a potential conflict of interest.

## Publisher's Note

All claims expressed in this article are solely those of the authors and do not necessarily represent those of their affiliated organizations, or those of the publisher, the editors and the reviewers. Any product that may be evaluated in this article, or claim that may be made by its manufacturer, is not guaranteed or endorsed by the publisher.
